# Knowledge- and ambiguity-aware robot learning from corrective and evaluative feedback

**DOI:** 10.1007/s00521-022-08118-z

**Published:** 2023-01-16

**Authors:** Carlos Celemin, Jens Kober

**Affiliations:** Cognitive Robotics Department, TU Delft, Mekelweg 2, 2628 CD Delft, The Netherlands

**Keywords:** Interactive imitation learning, Human reinforcement, Corrective demonstrations, Active learning, Uncertainty

## Abstract

In order to deploy robots that could be adapted by non-expert users, interactive imitation learning (IIL) methods must be flexible regarding the interaction preferences of the teacher and avoid assumptions of perfect teachers (oracles), while considering they make mistakes influenced by diverse human factors. In this work, we propose an IIL method that improves the human–robot interaction for non-expert and imperfect teachers in two directions. First, uncertainty estimation is included to endow the agents with a lack of knowledge awareness (epistemic uncertainty) and demonstration ambiguity awareness (aleatoric uncertainty), such that the robot can request human input when it is deemed more necessary. Second, the proposed method enables the teachers to train with the flexibility of using corrective demonstrations, evaluative reinforcements, and implicit positive feedback. The experimental results show an improvement in learning convergence with respect to other learning methods when the agent learns from highly ambiguous teachers. Additionally, in a user study, it was found that the components of the proposed method improve the teaching experience and the data efficiency of the learning process.

## Introduction

Learning the solutions for sequential decision-making problems in robotics has become a deeply studied alternative to traditional control engineering approaches. Leveraging the powerful capabilities of machine learning (ML) methods, it is possible to bypass most of the analytical and empirical work a skilled engineer should perform. Most of the research and big successes in this regard have been obtained through autonomous learning schemes such as reinforcement learning (RL) [1–5].

However, despite the impressive achievements, RL suffers from limitations regarding data inefficiency, the safety of the system, and the difficulties of reward engineering. The former limitation is especially a problem for learning with robots since more computational power does not fully solve the problem, while the latter is not so frequently discussed, and is an underrated problem in the literature because most of the recent developments have been evaluated in well-standardized benchmarks that already include a defined reward function.

Imitation learning (IL) [6] is a more direct learning approach that benefits from the knowledge of a teacher who demonstrates how to perform a task (i.e., provides a dataset of samples), instead of hand coding the required behaviors. After recording the demonstrations, a policy model is trained in order to imitate that dataset either with behavioral cloning (BC) [7, 8] or with inverse reinforcement learning (IRL) [9].

Interactive imitation learning (IIL) [10] expands the possibilities of IL approaches. With IIL, policies are learned incrementally while the teacher is in the learning loop providing feedback that improves the knowledge of the agent in every new situation, which leads to obtaining higher-quality data compared with only demonstrating the execution of the task. The feedback provided on top of the executions of the learning policy reduces the issues related to the distribution shift [11, 12].

Additionally, these interactive methods enable users to transfer their knowledge with other modalities of interaction, not only explicitly showing what the agent should do, but also guiding with evaluative feedback from the teacher, i.e., reinforcements (rewards or punishments), or also by comparing the performance of different agents/policies with learning from preferences or rankings. The use of evaluative feedback bridges the worlds of RL and IL [13–16].

Depending on the kind of task to be solved, and the expertise of the teacher in solving or understanding it, some of those modalities can be more convenient. Showing the agent what to do when it makes a mistake is the most efficient way to correct a policy; however, this requires that the teacher understands the dynamics of the environment well and knows a good strategy to achieve the goal of the task. Providing evaluations of the policy or comparing different policy executions enables less expert users to teach a robot, since they do not need to know what should be done, rather just to have enough insights about what is good/bad or what is better/worse. However, human reinforcements and preferences are kinds of feedback that contain less information than demonstrative feedback; therefore, methods based on them tend to be less data efficient.

Nevertheless, the choice of the preferred feedback modality is something that could change within the same learning process of a task. There could be situations wherein users are less skilled to demonstrate the right action than in some others. Also, it could happen that after some training time, users experience tiredness that leads to loss of concentration and engagement, consequently, having less insights about the right demonstrations, which could open the possibility of providing less demanding feedback, and leveraging human reinforcements.

**Fig. 1:**
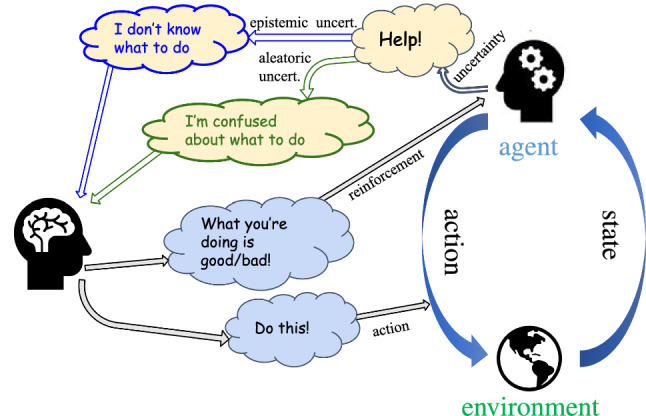
Interactive imitation learning with corrective and evaluative feedback, and active queries based on epistemic and aleatoric uncertainty

This work introduces ICREATe (Interactive Corrections and Reinforcements for an Epistemic and Aleatoric uncertainty-aware Teaching), a method that enables users to train agents with corrective demonstrations and evaluative feedback interactively in a Data Aggregation (DAgger) scheme, while employing the two kinds of uncertainty for active learning (Fig. 1).

The proposed method ICREATe improves the human–robot interaction experience via active learning (active queries from the robot to the user). The method endows robots with the capabilities of predicting the uncertainty of the policy in order to notify the teacher when the robot is not confident about the action to be executed. However, unlike previous works, two uncertainties are considered for the active queries: i) epistemic uncertainty, which indicates the lack of knowledge/data, and ii) aleatoric uncertainty, which considers the noise in the observations, produced by inconsistent or ambiguous feedback signals. Moreover, the system improves the data efficiency, aggregating implicit rewarded feedback in situations wherein teachers’ silence could be considered as positive rewards, for instance, when teachers do not correct actions and let the robot execute them despite it signaling uncertainty.

During training time, the teachers could decide anytime either to reward/punish the agent or to correct the policy with an action demonstration, both intermittently. Both kinds of feedback are smoothly combined to modify the same policy model, affecting the probability of choosing the actions to be taken.

Unlike previous works based on data aggregation, the use of aleatoric uncertainty-based queries allows avoiding the assumption of perfect teachers. Extensive evaluations with noisy simulated and real teachers show that this method can be widely applied in many scenarios with non-expert teachers, while still being able to learn high-performance policies in both simulated and real robot systems, and improving the user experience.

## Background

### Markov decision processes

Most of the RL and IL methods are based on the Markov decision processes (MDP) framework [1]. In this framework, the agent executes actions *a* in order to control the situation of the environment described by the state *s*. Those actions are executed in order to make the transitions of the state follow the objective of the task, represented by the reward *r*. The policy $\pi$ is the model that maps from states to actions, and the optimal policy is the one obtaining the maximum accumulated discounted reward. The dynamics of the environment are described by the transition function *T*, mapping from the current state $s_t$ and the action $a_t$ executed in that state to the next state $s_{t+1}$. The transition functions in MDPs feature the Markovian property, which means the transition to state $s_{t+1}$ depends only on the current state $s_{t}$ and action $a_t$. With the Markov property, the decisions are only a function of the current state in every time step, computed with the policy $\pi (s_t)$.

### Learning from human input

There are many methods for learning policies from experience and human teachers in the learning loop. The main two modes of user interaction with a policy learning agent are with the teacher providing corrective demonstrations, or with human reinforcements [10].

#### Learning from human reinforcements

The difficulties for reward engineering in RL have motivated to explore the possibility of replacing or complementing the MDP encoded reward function with human teacher reinforcements or evaluations. Seminal works in this direction evaluated to replace the reward function with the rewards a human teacher would provide in an interactive learning fashion, within standard RL methods [17–19].

However, human rewards have different considerations regarding past, present, and future, as regarded by the Bellman optimality [1] within MDPs. Some works have studied the intentions behind human reinforcements [20, 21], which have been useful for adapting the learning methods to human teachers.

Some works have proposed to learn from these human evaluations while using them directly to influence the decisions exclusively in the situation in which they were given, i.e., in the state that is rewarded. In Policy Shaping [22], the human evaluation is used for updating the probability of choosing the action $a_t$ the agent executed in the state $s_t$.

Training an Agent Manually via Evaluative Reinforcement (TAMER) [14, 23] is a framework that also considers the feedback for directly influencing only the action that is being executed in the corresponding state (if the human response delay is disregarded), without using it for computing a return. It assumes the feedback is directly conveying the desirability of the action and could be taken directly as a value rather than a reward. Therefore, the human reinforcement *h* is used for learning a Human model *H*(*s*, *a*) that tries to predict that human signal. This model is used for computing the policy with $\arg \max _a H(s_t,a)$, choosing the most desirable action according to the feedback *h* given by the teacher, as shown in Alg. 1
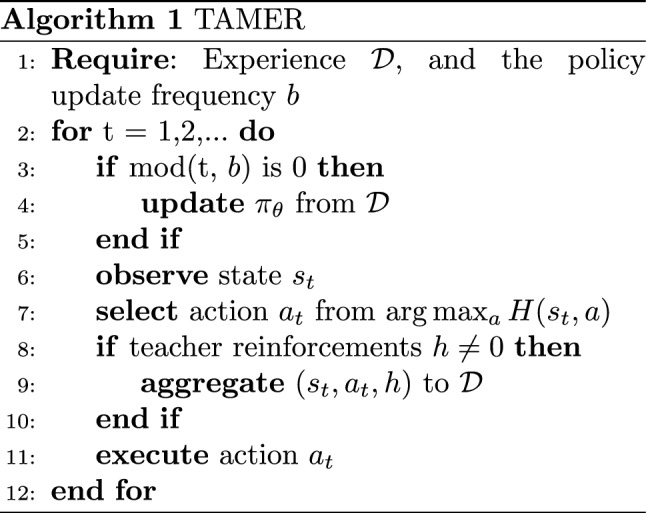


#### Learning from corrective demonstrations

In these methods, the human teacher is observing the agent performing the task, and whenever a correction is required the teacher can intervene for demonstrating the right action that the agent records and uses for updating the policy.

Confidence-based autonomy [24] was one of the seminal works in this direction, which models the policy with a Gaussian mixture model (GMM) used also for estimating the confidence of the policy. Whenever the policy is not confident about the action to take in the visited state, it can actively request a demonstration from the teacher that is incorporated in the data for learning the policy.

A very well-known IL method in which the teacher demonstrates the correct actions while the agent keeps following the current policy is Data Aggregation (DAgger) [25], which has shown to be powerful for reducing compound errors, given the distribution of the collected data is controlled by the learning policy. It has motivated the development of a family of methods SafeDAgger [26], EnsembleDAgger [27], LazyDAgger [28], or ThriftyDAgger [29], which deal in a different way the teacher interventions in order to improve feedback efficiency or safety.

One of those variations is Human Gated DAgger (HG-DAgger) [30], which was proposed for considering human teachers whose feedback quality could be degraded when not directly observing the effect of the actions they demonstrate. With this method, the teachers do not need to demonstrate every time step, but only when they deem a correction necessary. In such a case, the teachers’ actions are executed by the agent instead of the learning policy, improving the safety of the system because when visiting risky states, the expert can take over and return the agent to safe states. Ensembles of neural networks (NNs) are used to model the policy, having the additional capability of estimating the uncertainty of the policy, which can be used for risk warnings. In Alg. 2, a summarized pseudocode of HG-DAgger is presented, wherein the policy is updated every *b* time steps or even every episode.
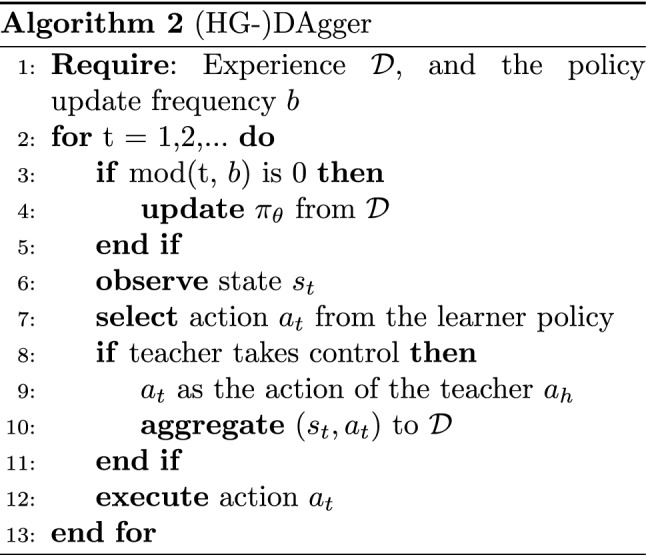


Research has found that there are clear trends in the preferences teachers have when training agents, regarding the kind of feedback to use [20, 31, 32]. However, no kind of feedback has shown to be better than all the others in all possible contexts, and each of them has a potential benefit in different situations. Hence, the possibility of using different interaction modes within one learning framework is an open challenge with good prospects for both the learning performance and the user experience, which has not been widely studied. Cycle of learning [33] is proposed to combine different modalities in a sequential manner, with different phases for each modality, and not in a simultaneous scheme wherein the teacher can choose what feedback to give at any moment. A combination of corrections and evaluations can be used simultaneously as proposed in [16]; however, the method works on top of a tabular RL method that can face scalability limitations.

Corrective and Evaluative Interactive Learning (CEILing) [34] proposes a smooth combination of corrective demonstrations and evaluations for problems of high dimensionality. Nonetheless, it does not make use of the entire spectrum of the evaluative feedback, since it processes only positive evaluations, but not negative ones. Therefore users cannot punish wrong behaviors, and they can only change them with corrective demonstrations, requiring the teachers to always be experts at the task execution.

This work proposes a framework that similarly aims to combine both types of feedback, but lets the user shape the policy with any feedback without restriction, i.e., being able to train policies even when using exclusively one of them, or both combined. Additionally, the learning agent is endowed with awareness of unknown states or ambiguous situations given the past feedback based on uncertainty estimation, which can improve the teacher’s performance.

### Types of uncertainty

In ML, it is very important to know how reliable is a model being trained. The loss function evaluated with the training, test, or validation sets is a global measure that can be taken as an index of the general reliability of the model, but it is not useful for explaining how reliable each prediction is.

Uncertainty estimation can provide more specific measures that can be used for understanding how trustworthy the prediction of the model is given an observation. The reliability of a model can be described as a composition of two different uncertainties: the *epistemic* and *aleatoric* uncertainties [35, 36].

*Epistemic* uncertainty explains whether the model has knowledge about the observed situation (input), i.e., this uncertainty is high when the model makes a prediction with an observation not seen in the training data. This uncertainty can be reduced by gathering more data that represents all possible situations. *Aleatoric* uncertainty explains how inconsistent the observations for training the model have been. It accounts for the noise in the data that makes it contradictory or ambiguous, for instance, when there is noise in the measurements, or specifically in the context of IL, when the teacher demonstrates contradictory actions for the same state. Aleatoric uncertainty cannot be reduced with the collection of more data.

Although each uncertainty models different issues, their use is not mutually exclusive [37]. A model can be considered reliable in predicting the underlying phenomena that generated the training data if it obtains a low estimation of both of these uncertainties. It means that very similar situations were observed during training (epistemic certainty) and that those similar situations did not introduce any inconsistency or ambiguity (aleatoric certainty).

Uncertainty estimation is important for knowing the reliability, and in some cases, the safety of the model when it is deployed. Nonetheless, it is also useful to estimate uncertainty while still learning, as it can support the process of finding the best learning samples by means of active queries. In IIL, various learning approaches have used uncertainty estimation for generating active queries [24, 30, 38, 39]; however, no method so far has focused on combining both uncertainties within an IIL method, such that the agent communicates to the teacher its awareness of unseen states and previous ambiguous interactions.

We propose to combine the prediction of both uncertainties within a data aggregation IIL scheme, helping to increase the users’ engagement with the learning process in critical situations. The proposed method is able to learn from the two kinds of feedback, but additionally, aleatoric uncertainty modeling is able to detect ambiguities in the feedback. This occurs not only when there are contradictory demonstrations or when there are contradictory evaluations, but also when there is feedback of a kind that contradicts feedback of the other kind, for instance, when in two different moments, for a specific state, an action is rewarded and another action is demonstrated or when an action is demonstrated and later the same action is punished.

## Interactive corrections and reinforcements for an epistemic and aleatoric uncertainty-aware teaching (ICREATe)

The IIL method ICREATe proposed in this paper enables a teacher to occasionally intervene in the learning loop by providing a feedback signal, whenever she/he considers the action being executed wrong, or in order to reinforce a correct behavior.

It is important that the learning agent helps to keep the teacher aware when it is required to correct the policy, at least when the policy is not confident. If there would not be an active query, the teacher could notice too late that feedback was required and might need to wait until the same situation happens again. Additionally, the agent could end up in a dangerous/undesirable state. Therefore, implementing active queries can help with improving the data efficiency of the learning process and the safety of the system.

In this method, the epistemic and aleatoric uncertainties of the policy are modeled such that the agent can convey to the teacher when it is not confident as: i) it is facing an unseen situation, i.e., visiting states wherein the teacher has not provided any feedback signal; ii) it is visiting states in which the teacher has given ambiguous/contradictory feedback signals. With these queries, the teacher can decide whether to provide evaluative or corrective feedback to the agent for either accepting, rejecting, or explicitly correcting the performed behavior.

ICREATe is composed of two main parts: (i) the integration of two different interaction modalities for the teachers to train the agents: corrective and evaluative feedback (Sect. 3.1) and (ii) the estimation of epistemic and aleatoric uncertainty to generate active queries when the policy is uncertain, to improve the teacher’s engagement with the learning process (Sect. 3.2). The combination of these two modules leads to an additional third component which is based on a passive positive feedback assumption that labels some of the state–action pairs that are not corrected or punished by the teacher with positive rewards, which is elaborated in Sect. 3.3.

### Learning from corrective and evaluative feedback

Teachers can share their insights about the policy execution through two different modes of interaction: corrective demonstrations or evaluative reinforcements. Either of the two interaction modes can be preferred at any moment depending on the complexity of the problem or the current transitions, the expertise of the teachers, their engagement, the performance of the learning agent, or some other factors.

The policy $\pi (a \vert s)$ predicts the probability of choosing the action *a* given the state *s*. Both kinds of feedback signals are directly used to modify the probability of choosing an action in the update of the policy model $\pi$. When there is a teacher intervention for providing a feedback signal, a triple (*s*, *a*, *h*) is aggregated to the training dataset $\mathcal {D}$, where $h_t$ is the evaluative signal. The way the feedback is parsed and used to generate the labels for training the policy with supervised learning depends on the kind of feedback the teacher provides as explained below:

#### Corrective feedback

The teacher can intervene to provide corrective demonstrations whenever considered necessary as the agent is performing wrong actions. Like in HG-Dagger, actions demonstrated by the teacher are not only recorded but also executed by the agent, in order to improve the safety in the environment, i.e., the intervention of the teacher is directly the gating function that selects to execute the action of the teacher instead of the one of the learner.

In this case, the teacher uses an interface that allows to take over and control the robot at any time. During the corrective demonstration at the time step *t*, the demonstrated action $a_h$ is assumed to implicitly receive a positive reward $h_t = 1$ since it is considered the right action; thus, the triple $(s_t, a_h, 1)$ is added to $\mathcal {D}$.

#### Evaluative feedback

The teacher can also intermittently provide an evaluation of the executed action. Unlike the corrective feedback that is executed during the current time step in which it is provided, the human reinforcements are an evaluation of the executed action $a_{t-1}$ after observing its effect in the transition from $s_{t-1}$ to $s_t$. Therefore, the evaluation is associated with the previous time step $t-1$, and the triple aggregated to $\mathcal {D}$ is $(s_{t-1}, a_{t-1}, h_t)$, where $h_t$ is 1 or $-1$ depending on whether the teacher reinforcement is a reward or a punishment, respectively.

#### From feedback to output labels generation

In order to train the policy in a supervised learning fashion, the triples stored in $\mathcal {D}$ are used for training the policy $\pi$ which maps from the states to the actions desired by the teacher. Since $\pi (a \vert s)$ computes the probability of choosing any action *a* given the state *s*, the method computes the target probability label based on the action *a* and the human evaluation *h* recorded in each triple.

If an action is either rewarded or explicitly demonstrated, the method will increase the probability of choosing that action, but if the action is punished by the teacher, the probability of choosing it will be decreased.

*Positive evaluations* If $h=1$, the vector of labels used for training the policy is a one-hot encoding, wherein the action with the high value is the one that was either demonstrated explicitly by the teacher, or the one that the robot executed, and the teacher rewarded positively. This label increases the predicted probability of that action in the policy.

*Negative evaluations* If $h=-1$, the label for all the actions could be set to zeros, which would mean that all actions are equally not desirable, including the ones not punished. However, doing that is a loss of information conveyed by the teacher, who implicitly means that *“the action punished is less desirable than some others”*; therefore, the probability of choosing this action should decrease more with respect to the others. Hence, it is proposed to set the target vector as a one-cold encoding that has the low value for the action that the teacher has punished. This decreases the probability of the punished action, while slightly increasing it for all the others.

*Label generation* From every triple (*s*, *a*, *h*) in $\mathcal {D}$, an input–output tuple $(s,l_i)$ is created for the supervised learning process, wherein $[ l_0, l_1, \ldots ,l_i,\ldots , l_C]$ is the vector of labels for the *C* possible actions, which are the components of the output of the policy.

1 $$\begin{aligned} l_i = L(a_i, a, h) ={\left\{ \begin{array}{ll} \mathbbm {1}\{a_i = a\}, &{} \text {if } h=1 \\ \mathbbm {1}\{a_i \ne a\}, &{} \text {if } h=-1 \end{array}\right. } \end{aligned}$$

This approach for label generation is convenient, especially for some kinds of teachers who tend to only punish wrong behaviors, while passively observing the good transitions, forgetting to reinforce behaviors with positive feedback. In this case, the actions that receive the least amount of punishments are the ones which the policy would assign the highest probability, i.e., this even allows to train a policy only using negative rewards that force the agent to try other actions, until the right one is found.

*Loss function* Non-one-hot encoding is used for multi-label classification problems minimizing the sum of multiple binary cross-entropy (BCE) loss functions. However, multi-label classification can consider and make decisions that are not compatible with the MDP framework, for instance, deciding on more than one action or none. In sequential decision-making problems, the policy is expected to decide on only one preferred action for every state. Accordingly, despite some of the *L* vectors not being a one-hot encoding, we propose to approach this problem as a multi-class classification using a categorical cross-entropy (CCE) loss, which additionally has the advantage of normalizing the output of the policy that can be interpreted as probabilities

2 $$\begin{aligned} \mathrm{CCE} = -\sum _{i}^{C} L(a_i, a, h) \log (\pi (a_i \vert s)). \end{aligned}$$

The softmax activation function is used to compute $\pi (a_i \vert s)$. Nevertheless, in preliminary experiments, we found that training in a multi-label scheme with BCE loss, or even using mean square error (MSE) obtains very similar results, although that is not the focus of the paper.

### Epistemic and aleatoric uncertainty estimation

In order to improve the engagement of the teacher with the learning process, ICREATe is endowed with the capability of actively letting the teacher know when it needs more data to improve its knowledge base. Due to different factors, teachers’ concentration and attention to the agent can decrease over time.

During the learning process, it is not always necessary that the teacher is completely focused on the task execution, especially when the policy has improved its initial performance. Indeed, when the agent starts to perform well most of the time, teachers tend to get distracted because mistakes are not expected anymore. Therefore, query generation based on policy uncertainty is a convenient strategy for increasing the teacher’s attention when it is more necessary. Both types of uncertainty are modeled independently as introduced in the following.

#### Modeling epistemic uncertainty

As implemented in previous works, we propose to use ensembles of NNs for capturing the uncertainty of the policy model [27, 30]. These models are composed of multiple NNs or heads of a NN, that are trained to predict the same output in each of them, given a specific input.

After training those models, ideally, all the outputs should agree with a similar prediction for the data used during training and the surrounding neighborhood, while the predictions of unseen states tend to have a high disagreement (variance). Additionally, the disagreement of the individual predictors of an ensemble has a positive impact on the ensemble generalization error [40].

The disagreement of the components of an ensemble is the lower bound of the weighted average of the errors of the components [41]. This means that if an ensemble predicts high disagreement for a specific state, it would obtain a high average error. This would only happen when that state is not similar to any sample of demonstrations used for training, and therefore, the disagreement can be interpreted as a measure of lack of confidence or uncertainty in the prediction of the policy, because more demonstrations are required for that situation.

In order to make an ensemble work well for predicting high variance for unseen states, there are some strategies that ensure that each of the heads of the NN is not trained exactly as a copy of the others, but rather generalizing in different ways while fitting the training data. Each of the components of the ensemble has a different structure that can be obtained with simple strategies like:

- Setting different depths for each component.
- Setting different widths for each component.
- Allocating different random batches for computing the updates of each component.
- Training under-regularized models.
- We also found it useful to randomly initialize the weights, forcing each output to start in different ranges, such that the initial predictions have a uniform distribution.

Although the action prediction of the policy and the epistemic uncertainty are both computed with the outputs of the ensemble, we simply name $\pi (s)$ the function that computes the $\arg \max$ of the average of the ensemble $\pi _E$,

3 $$\begin{aligned} \pi (s) = \underset{a_i}{\arg \max } \sum _{k}^{K} w_k \pi _{E,k} (a_i \vert s) \end{aligned}$$

where *K* is the number of individual predictors or heads of the ensemble and $w_k$ are optional parameters that are proportional to the performance of each head that are used for a weighted average of the outputs. The epistemic uncertainty is obtained with the function *G*(*s*) that computes the variance of the ensemble $u_e = G(s)$. Each of the heads of the ensemble $\pi _{E,k}$ is trained optimizing the cost function (2)

#### Modeling aleatoric uncertainty

In order to detect the noisy samples in the gathered data, which creates conflicts or ambiguities during training, we propose to train a predictive model *A*(*s*) that computes the probability of the policy predicting a mistake. This measure is inspired by the training of models for residuals prediction, which predict the error of the actual model (policy), with respect to the state. However, our interest is not to be able to predict the error of the policy, but rather to detect the states in which the policy has a prediction error.

The assumption is that the policy model is expressive enough to be able to imitate all the demonstrated actions after enough training, and only when there are conflicting/ambiguous demonstrations, the policy will have issues predicting the demonstrated action for the inputs of the demonstrations involved in the conflict.

For instance, if the teacher demonstrated two or *N* different actions for the same state *s*, the policy will be able to predict the action that is correct at most for one of those demonstrations, while not imitating correctly at least one or $N-1$ cases, respectively. In other words, we propose that the aleatoric uncertainty $(u_a = A(s))$ is indirectly measured based on the probability of predictive mistakes of the policy that are produced due to the ambiguous demonstrations and that cannot be solved with more training, but with more feedback that reinforces one of the possible actions, reducing the average error for that state.

The ideal prediction assumption is not always realistic, especially at the beginning of the learning process, when the model is not fitting all the training data. Nevertheless, the prediction of mistakes in non-ambiguous states is not a negative feature, but rather a good side effect that helps to request feedback in states that need to be emphasized because the demonstrated data is not being imitated yet. For training this model *A*(*s*), the update procedure is performed subsequently after the update of $\pi (s)$.

*Predicting mistakes when only learning from corrective demonstrations:* For each demonstrated tuple (*s*, *a*) in the update batch, the target *m* (for the model *A*(*s*)) for the input *s* is computed as

4 $$\begin{aligned} m = M(s,a) = \mathbbm {1}\{ d(a,\pi (s)) > \epsilon \}, \end{aligned}$$

where $d(\cdot )$ is a measure of distance/similarity and $\epsilon$ is the threshold defining whether two actions are similar or not, depending on the domain of the actions. For the case of discrete actions, the target of the tuple (*s*, *m*) can be computed as

5 $$\begin{aligned} m = M(s,a) = \mathbbm {1}\{ a \ne \pi (s) \}. \end{aligned}$$

*Predicting mistakes when learning from corrective and evaluative feedback:* For this more general case approached in this paper with the proposed method, the generation of the output label of *A*(*s*) is slightly more complex since there are samples of undesirable state–action pairs, i.e., when $h = -1$. The variation of *M* in (6) takes the triple (*s*, *a*, *h*) to compute the output $m= M(s,a,h)$ associated with the input *s*, conditioning it with the evaluative feedback *h*.

6 $$\begin{aligned} m ={\left\{ \begin{array}{ll} \mathbbm {1}\{ a \ne \pi (s) \}, &{} \text {if } h=1 \\ \mathbbm {1}\{ a = \pi (s) \}, &{} \text {if } h=-1 \end{array}\right. } \end{aligned}$$

**Loss function:** This supervised learning problem is trained optimizing the BCE loss computed with:

7 $$\begin{aligned} {\begin{matrix} BCE = - {}M(s,a,h) \log (A(s))\\ \qquad\qquad\qquad\qquad\, -(1 - M(s,a,h)) \log (1 - A(s)) \end{matrix}} \end{aligned}$$

#### Network architecture

**Fig. 2:**
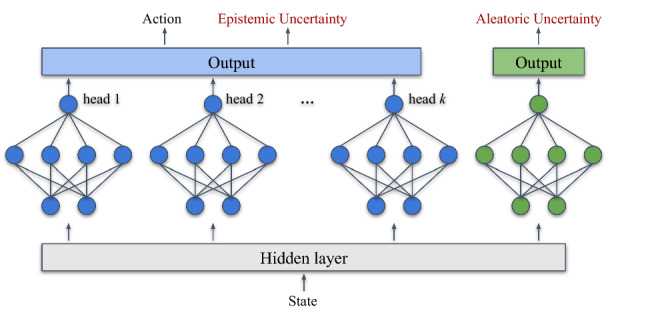
Network architecture for predicting actions, along with the epistemic and aleatoric uncertainties

We propose to implement one big network as presented in Fig. 2, that includes the ensemble for computing the policy $\pi (s)$ and the epistemic uncertainty with *G*(*s*), along with the mistake prediction model *A*(*s*). It has a hidden layer (in gray) after the input layer, which is common and connected to all the heads of the network, including the branch for computing *A*(*s*) (in green). This is with the purpose of sharing the first set of feature extraction such that not everything is learned completely independently and the process is more data efficient.

The layers of the policy ensemble are in blue, and each of the heads has a softmax activation in the layer connected to the output layer, such that for each head the cost (2) can be minimized independently. The last blue output layer is for computing the variance of the ensemble *G*(*s*) and the average of (3) for $\pi (s)$.

The green head contains the layers for computing *A*(*s*), and its output layer has a sigmoid activation function required for computing the binary cross-entropy loss (7).

### Passive rewarding under uncertainty

Teachers do not tend to perform corrective demonstrations or provide positive rewards all the time when the agent is executing the right actions. There are situations in which there were ambiguous demonstrations but the action predicted by the policy is considered satisfying by the teacher, or unseen situations with high epistemic uncertainty in which the actual prediction is also the desired action.

For those cases, some teachers would decide to not always provide a feedback signal, especially after a reasonable training time, even though there is a query/alert triggered by high uncertainty estimations. The results of [42] support the hypothesis of considering silence as positive feedback.

We propose to generate more training data even without the active intervention of the teacher, leveraging the aforementioned assumption. Since the teacher is aware of the low confidence of the policy due to the active queries, we assume that the lack of teacher interventions with either corrective demonstrations or punishments could be taken as an implicit acceptance or approval of the current executed behavior, unless the teacher lets the agent know that she/he will not provide feedback. Therefore, the state–action pairs of these situations are considered for this passive rewarding and aggregated to the dataset with a reinforcement $h=1$.

Nevertheless, it is proposed not to passively reward every time step there is a query and the teacher does not intervene. Rather, it is taken into account that teachers could have a slow reaction to new events, and hence, a grace period should be considered before the passive rewarding. In [43], this response time has been modeled with probability distributions *P*(*t*) that explain how long it takes for a person to react to different tasks, or the probability of having a reaction after *t* seconds of the event.

In order to make sure the algorithm does not passively reward state–action pairs before the probabilities of response are reduced significantly, the grace period is calculated from those models finding the time in which there is only $5\%$ left of probabilities of response, i.e., using (8) to find $t_g$.

8 $$\begin{aligned} \int _{0}^{t_g} P(x) \,dx = 0.95 \end{aligned}$$

In [14], an example of this response time is shown, wherein $P(t) \sim {\text {Gamma}}(2, 0.28)$, and finding $t_g$ from (8) is approximately 1.3*s*, which matches the experimental results presented in [43]. Thus, the passive rewarding is performed only when there are continuous queries from the agent for more than $t_g$ seconds without teacher intervention, unless the teacher disables it with:

- A signal that sets the teacher interaction on hold. This is useful for allowing the teacher to perform another task, as long as the system does not have any safety-related risk.
- A signal that states the teacher does not know whether the robot execution is right in the current time steps.

The passive rewarding continues every time step after $t_g$ as long as the conditions do not change; otherwise, it is interrupted and the time counter would be restarted when those conditions are fulfilled again.

### Complete ICREATe algorithm

The integration of these components considering the two interaction modes, the use of both uncertainty estimations for active learning, and the passive rewarding component is presented in Alg. 3. The parameters $\theta$ and $\phi$ comprise the networks of the policy ensemble and the aleatoric uncertainty model, respectively; therefore, the implementation of the parameterized functions is named $\pi _\theta$, $G_\theta$, and $A_\phi$.
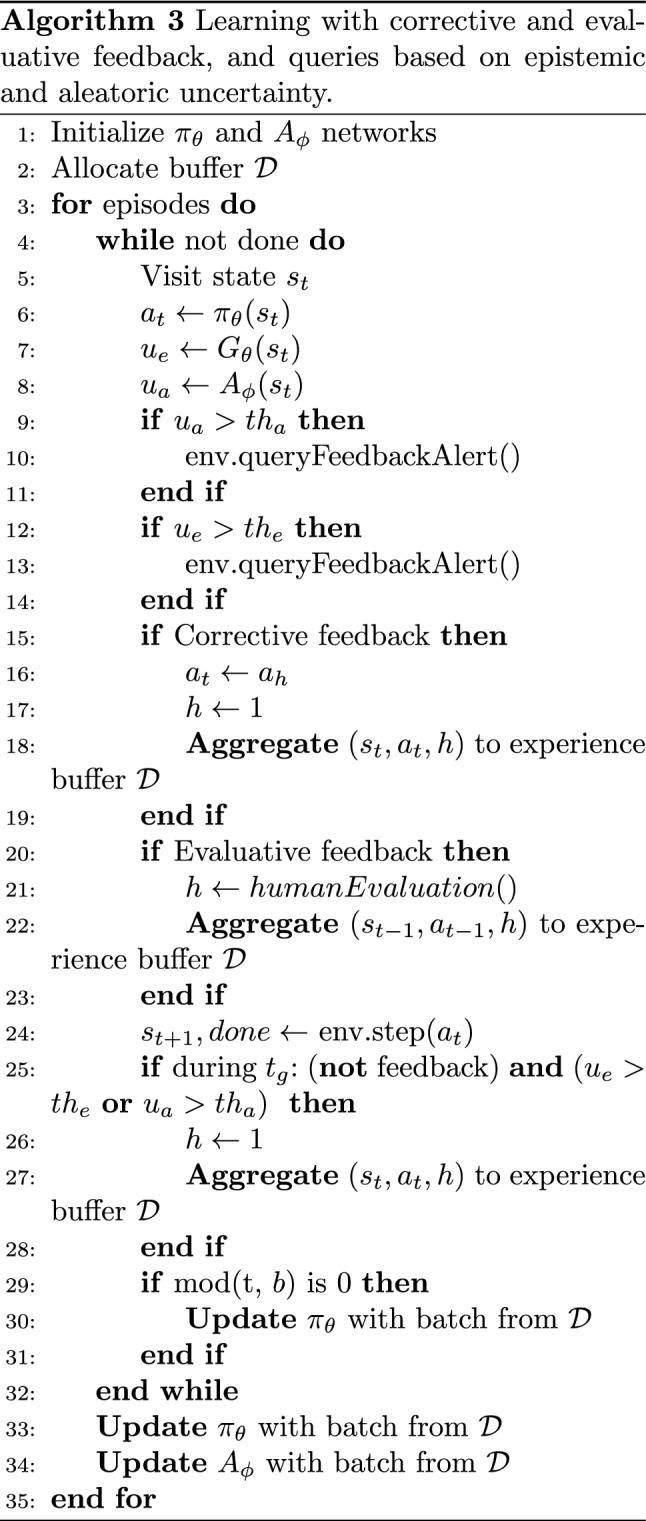


For every episode, in every time step, the policy and uncertainty models are evaluated in $(s_t)$ (lines 6–8). In order to generate active queries, the uncertainties $u_e$ and $u_a$ are compared to the thresholds $th_e$ and $th_a$, respectively, and if *True*, the function *env*.*queryFeedbackAlert*() is used to communicate the query to the teacher through the user interface (lines 9–14).

If the teacher decides to intervene with a corrective demonstration (line 15), the action $a_h$ demonstrated by the teacher with the user interface replaces the current action of the policy $a_t$ (line 16), and it is used with the default reinforcement (line 17) to be aggregated to $\mathcal {D}$ (line 18). If the user decides to intervene with evaluative feedback, the function *humanEvaluation*() obtains the reward or punishment from the user interface (line 21); it is aggregated to $\mathcal {D}$ (line 22). Then, the action $a_t$ is executed by the agent (line 24).

The passive rewarding is computed (lines 25–28), if all the requirements discussed in Sect. 3.3 are fulfilled. In line 25,“not feedback” means that neither a corrective nor evaluative feedback has been provided by the teacher.

Finally, the policy is updated every *b* time steps. At the end of every episode, both $\pi _\theta$ (implicitly $G_\theta$) and $A_\phi$ are updated with loss functions (2) and (7), respectively. This algorithm can be seen as a generalization of some other methods that can be derived from ablations of Alg. 3. For instance, HG-DAgger is obtained if only running the lines 1–7, 12–19, 24, and 33. Deep TAMER would be only with lines 1, 3–6, 20–24, and 29–33 along with removing the softmax activation function layer. The source code of this method will be published along with this paper.

## Experiments and results

In order to evaluate ICREATe, in the experiments, it was considered both simulation and real robot setups, along with evaluations including real and simulated teachers.

### Experimental setup

The evaluation of ICREATE was planned sequentially, first taking the most exhaustive comparison using simulated teachers or oracles to replace the human in the loop, then running a user study with a simulated environment, and finally a validation with tasks with a real KUKA iiwa robot arm.

#### Simulated environments with simulated teachers

For simplicity, the most exhaustive experiments are carried out with simulated environments since they do not have physical constraints, like the duration of the experiment (that cannot be accelerated with computational power), or the safety of the system itself and the users around.

**Fig. 3:**
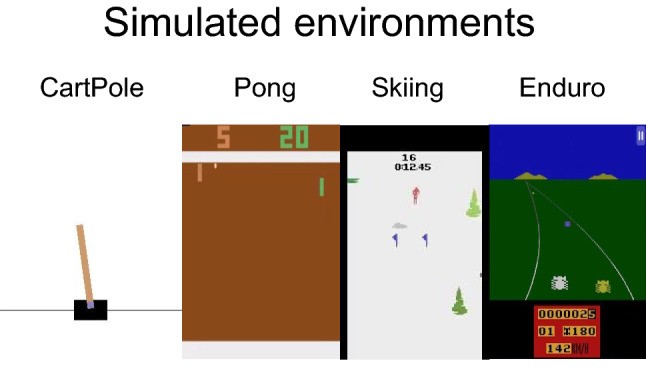
Simulated environments used for experiments

*Environments:* Four different OpenAi Gym [44] environments were chosen for these evaluations, considering different complexities with low-, intermediate-, and high-dimensional state spaces. For a low-dimensional problem, the CartPole environment is used, which has a very simple state space of four dimensions. With an intermediate dimensionality, the Atari games Skiing and Pong are used, specifically the environments using the RAM memory as observation vector with a length of 128, which is not a so high-dimensional observation, but still, a complex representation from which it is difficult to obtain good features extraction, i.e., it is a representation that requires smaller NNs and less computational resources with respect to using images as observations, but in some cases, it is less efficient to decode the relevant information than when learning with pixels in the observations. For high-dimensional observations, the Enduro environment with screen images as observation is used, and the last four frames are considered within the current observation. Figure 3 shows screenshots of the chosen Gym environments.

*Simulated teachers:* In order to evaluate and compare different methods without biasing the experiments with human factors like tiredness or loss of concentration after many repetitions of many different tested methods, or the influence of the order of the used teaching methods, a simulated teacher based on an expert policy is implemented. It is able to perform many learning processes consistently without external human factors allowing to purely evaluate the algorithmic potential—measuring how each algorithm is able to make the learner imitate the teacher, based on the velocity of convergence and final performance.

Depending on the task, the oracle is set to provide feedback to the learner on a fixed percentage of time steps, based on preliminary experiments. For each task, it was measured what percentage of time steps an expert teacher gives feedback, and additionally how many episodes of feedback are required for the oracle to provide feedback to the agent, such that the learning agent reaches a good performance. The rate of feedback and the number of episodes with feedback are fixed for each environment, for running all the experiments under similar conditions.

In the case of passive learners, the simulated teacher provides feedback randomly with the predefined rate of feedback, whereas for the active learners the random feedback is controlled in order to compensate for the number of time steps wherein the learner requested feedback. In the cases of using both corrections and evaluations, the probabilities of providing either are set arbitrarily to $50\%$. For the corrections, the expert policy is evaluated in the visited state $s_t$ and using it as $a_h$. But for evaluative feedback, the reinforcement is obtained as in (9), comparing $a_t$ computed with the current policy $\pi _\theta (s_t)$ to the action $a_h$ computed by the oracle.

9 $$\begin{aligned} h ={\left\{ \begin{array}{ll} 1, &{} \text {if } a_h = \pi _\theta (s_t) \\ -1, &{} \text {if } a_h \ne \pi _\theta (s_t) \end{array}\right. } \end{aligned}$$

Since the interest of this work is in interactive imitation learning approaches that can deal with ambiguous or noisy human feedback, all the experiments with simulated teachers incorporate simulated mistakes in the feedback. Actually, the mistakes are a variable that cannot be controlled or observed in experiments with real users; therefore, the robustness of the methods to that factor is only studied in this set of experiments with oracles.

For all the experiments, the percentage of mistakes is set to $40\%$, which means that $40\%$ of the time steps the oracle intervenes, it provides wrong feedback. In the case of a correction, it selects a random action out of the set of actions excluding the right one, whereas in the case of evaluations it provides a reinforcement contrary to (9). This rate of mistakes is very high since almost half of the interventions of the teacher are wrong. This high rate of mistakes is intended to assess how the use of aleatoric uncertainty-based queries improves the robustness of the learning process.

*Ablations* For comparison purposes, ICREATe and many ablations of it which match other state-of-the-art interactive methods such as HG-DAgger [30] and deep TAMER [23] were evaluated, along with variations/extensions of them, considering additional features such as the active learning components and updates during/after episodes. Also, the CEILing method [34], which combines corrective and evaluative feedback, was included in the experiments.

The generated algorithms and ablations employ (or not) the following different algorithmic features:

- **AQ:** Active queries based on aleatoric uncertainty.
- **EQ:** Active queries based on epistemic uncertainty.
- **Episodic update:** The policy only updates every episode, otherwise every *b* time steps ($b=1$ in the implementation).
- **Passive reward:** Use of the passive rewarding under uncertainty assumption.
- **Corrections:** Use of corrective feedback.
- **Evaluations:** Use of evaluative reinforcements.

The last two could be considered algorithmic or oracle variables. Additionally, there is another considered feature that is not based on an algorithmic variable, but on the implementation of the oracle, i.e., this feature does not generate more variations of algorithms. This feature considers to reduce the rate of mistakes of the oracle to zero in time steps where the agent queries feedback. It is based on the assumption that a human teacher might do fewer mistakes whenever the agent requests feedback, because it improves the engagement of the user.

At the beginning of the learning process, most of the queries are due to epistemic uncertainty since most of the state space is unseen, i.e., there are not many collected labels yet. And since there are not many labels, it is unlikely there are many ambiguous/conflicting labels that generate aleatoric uncertainty-based queries. Therefore, we consider this variation only for the algorithms using queries based on aleatoric uncertainty, because those are the queries that keep being triggered in the long term during late episodes, that is, when the user might be tired and could get more distracted, and therefore, queries can have more influence (queries may not have a considerable impact at the beginning when the teachers are still focused).

The assumption of teachers not making mistakes because there is a query is not very strong, because users could still get confused or change their minds after a query. Nevertheless, couples of ablations that share the same features and differ only in the mistakes-related feature can provide a rough estimate of upper and lower bounds for the range wherein a real user could perform.

With all the seven features that can be considered (or not), 128 different variations could be generated, although a few of them do not make sense to be implemented. There still are an unfeasibly high number of possible experiments that could be carried out. Therefore, for simplifying the experiments to a doable and reportable scale, only the variations that are closer to the original algorithms are selected, as reported in Sect. 4.2.1.

#### User study

Unlike other non-interactive machine learning methods, the evaluation of human-in-the-loop approaches requires not only to assess the capability to converge to successful results, but also the impact they have on the user experience. To complement the objective measures that can be obtained with the simulated teachers’ experiments, a user study that collects subjective measures is carried out in order to observe how variations of the proposed method improve the experience of the users.

In previous studies, it has been shown that users tend to prefer teaching by showing what to do rather than evaluating what the learner is doing. Hence, user studies for comparisons of learning with different kinds of feedback are not going to obtain very new conclusions. The focus is rather on evaluating the use of both uncertainties for active learning. Five different variations of the proposed learning approach are used in this study; in all cases, users can teach with both evaluative and corrective feedback. The evaluated systems feature:

- **PL:** Passive learning, no use of queries.
- **EQ:** Queries based on epistemic uncertainty.
- **AQ:** Queries based on aleatoric uncertainty.
- **EAQ:** Queries based on epistemic and aleatoric uncertainty.
- **EAQPR:** Queries based on epistemic and aleatoric uncertainty, along with the passive rewarding strategy.

The participants of the experiments were requested to answer simple and short questions from well-known questionnaires for assessing user experience. They completed the System Usability Scale (SUS) questionnaire [45], which obtains a score of usability for each of the systems, along with the NASA-TLX [46] that measures the perceived workload of the systems (in this case the raw TLX [47] is used), and the system acceptance scale [48], which measures scores for usefulness and satisfaction of the system.

Additionally to these subjective measures, the final average performance of the obtained policies and the amount of feedback the teachers provide to the learning agent is also collected from the learning processes. This count of human corrections and reinforcements is presented raw instead of as a percentage of time steps. The reason is that policies with a lower performance result in a longer episode duration in the Skiing environment; therefore, a low percentage could hide a high amount of interactions in long episodes.

For the experiment, the previously introduced Skiing game from Atari is used, since, in our observations, it is a difficult learning challenge that can get a lot of progress within a short period of time, something that is important for not losing the motivation of the participants. Additionally, this environment is a good motivation for IIL as the literature shows that it is very difficult for RL algorithms, which obtain poor results in general, given the sparsity of its environmental reward, whereas it is more direct to learn from the insights shared by a human teacher.

In the experiments, the participants listen to the guidelines and the instructions to follow; then, they are given five minutes to play with the environment. The order of the system they interact with is chosen randomly for each participant. They train the agent for ten minutes with every system and then immediately proceed to answer the questionnaires after interacting with each of them.

**Fig. 4:**
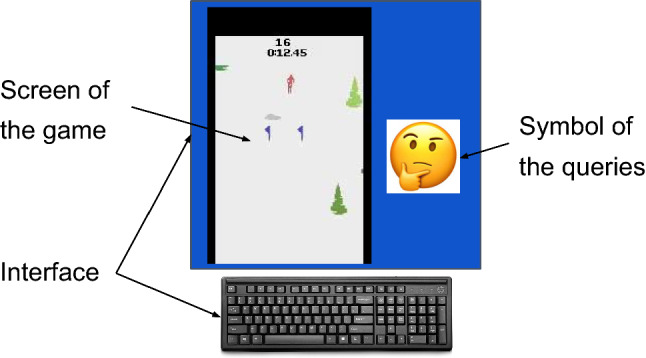
Interface for the user study

The participants sit in front of a computer wherein they observe the game on the screen while providing feedback through a keyboard as depicted in Fig. 4. Beside the screen of the game, there is a dark empty window that displays a smiley any time the system is querying feedback.

In these experiments, the participants did not have any technical background in robotics and/or machine learning, featuring 8 men and 6 women, whose ages ranged between 24 and 55 years. The protocols of these experiments were evaluated and approved by the Human Research Ethics Committee of TU Delft. The participants signed an informed consent for joining this experiment.

#### Real robot environments

**Fig. 5:**
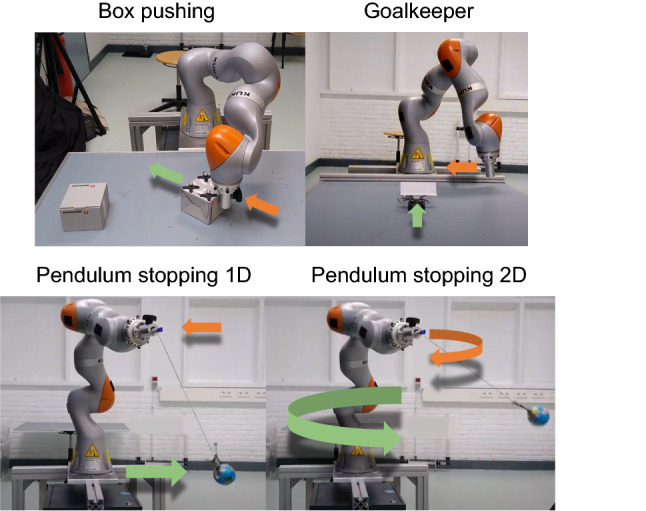
Setups for the validation with a physical robot

A validation of ICREATe in a physical robotic system is carried out with a KUKA iiwa 7 robot arm, with four different manipulation tasks introduced in the following and whose setups are shown in Fig. 5.

*Box pushing:* The robot has to push a box to be placed beside the box on the left side of the table, with the same orientation. The box is initially located with a random pose on the right side of the table. The episode finishes successfully if the box reaches the desired pose; otherwise, it is terminated unsuccessfully after 2 min. The objective function is the success rate.

*Goalkeeper:* The robot moves on a straight line (from the left to the right side of the table in Fig. 5) in order to intercept an object that moves toward this line (from bottom to top). The object starts the episode (at the bottom) with a random position and orientation, if it crosses the line of movement of the robot, it is considered a failed episode (goal) and otherwise a successful one when the robot intercepts the object. The objective function is the success rate.

*Pendulum stopping 1D:* A ball is attached to the robot end-effector with a rope, and the objective is to move the robot such that it compensates for the swing of the ball, reducing its velocity to almost completely stop the swing. At the beginning of the episode, the ball is placed higher than the end-effector and let fall free to start swinging. The ball swings through the same straight line in which the robot moves (from left to right). The objective function is the negative of the time the robot takes to stop the pendulum (a threshold of minimum velocity is assumed for considering the pendulum to be stopped).

*Pendulum stopping 2D:* The task is similar to the previous one, but in this case, the robot moves in a horizontal plane, and the ball is pushed to swing in circles.

For all the tasks, the actions are the change of position of the robot end-effector, whereas the states are composed of the positions and velocities of both the end-effector and the manipulated object. For the box pushing task also, the orientation of the box is considered.

In these setups, the states are obtained from the internal sensors of the robot and an OptiTrack motion capture system. The interface for providing feedback to the agent is a gamepad, and the participant of the experiment is an expert teacher.

### Results

#### Experiments with simulated teachers

As mentioned in Sect. 4.1.1, it is not possible to experiment with all the possible ablations and methods; therefore, the most meaningful ones were implemented, and from those, only the most interesting results are reported. The results of the learning convergence are summarized with the final performance of the policies, and the approximate number of episodes required for that convergence. Some ablations are considered a variation of an original algorithm if there is a change in the algorithmic variables, and not in the implementation of the oracle.

All the evaluated cases of ICREATe were tested in couples considering both mistakes and no mistakes during AQ. Additionally, almost all its variations were tested with the oracle: (i) providing $50\%$ of corrections and $50\%$ of evaluations and (ii) providing $100\%$ of corrections and no evaluations. The latter is to observe the performance when using the most efficient feedback mode, in terms of data efficiency, the best case scenario.

In the case of the CartPole results in Table 1, first, it is observed that none of the passive learners could get a relative progress in the learning process. The full ICREATe method was tested (results in rows 15–18 of Table 1), and two variations of it were also evaluated. The first variation (ICREATe V.1 in rows 9–12) did not include the passive rewarding assumption, obtaining a performance reduction between approximately 3–30% with respect to the full method. The second variation (ICREATe V.2 in rows 13–14) did not include passive rewarding and additionally updated the policy every time step. This variation decreased the learning policy performance even more, reducing it between 4 and 10% with respect to ICREAte V.1 (the corresponding cases in rows 9–10) and 13 and 39% with respect to the full method (the corresponding cases in rows 15–16). Because of these results, the second variation was not evaluated for the cases with $100\%$ corrective feedback. It is interesting to notice that with the full ICREATe and only using corrective demonstrations, the performance was slightly decreased with respect to also using evaluative feedback, being in both cases close to the optimal performance. In general, ICREATe outperformed D-TAMER, HG-DAgger, Ceiling, their variations, and the ablations of ICREATe itself, confirming that all the components of the method have a contribution in the improvement.

Five variations of D-TAMER were tested along the original, observing that unlike data aggregation schemes, D-TAMER performs better when the update is more often during the episode and not only at the end. As expected, having both uncertainties for active learning in this method obtained the highest performance. The original HG-DAgger along a variation that updates the policy every time step were tested (rows 7–8), showing that data aggregation methods collect better data and converge better when using episodic updates (as seen with ICREATe V.1 vs V.2). Finally, CEILing was tested twice, with “CEILing 3x” receiving oracle feedback three times more often than in the rest of the tested algorithms. However, increasing the amount of feedback had a slight effect. In general CEILing showed to be very sensitive to the simulated mistaken feedback.

For the environments of Skiing, Pong, and Enduro, only the most relevant cases from the experiments of CartPole are presented in Tables 2, 3, and 4, respectively. The same trends in these experiments can be observed, noticing that in these cases unlike in CartPole, there is always a positive and higher improvement when using only corrective demonstrations than when combining both feedback types. This is more noticeable because in these problems the action space dimensionality is higher; therefore, the difference in the amount of information contained in a corrective demonstration compared with an evaluative reinforcement is also higher.

**Table 1:** Results of the simulated teacher experiments with the CartPole environment

		Algorithmic variable				Algorithmic or Oracle variable		Oracle variable	Convergence	
#	Method	AQ	EQ	Episodic update	Passive reward	Corrections	Evaluations	No mistakes in AQ	Return	Episodes
1	D-TAMER	n/a	n/a	n/a	n/a	n/a	x	n/a	24.4	175
2	D-TAMER V.1			x	n/a	n/a	x	n/a	19.25	85
3	D-TAMER V.2		x		n/a	n/a	x	n/a	82.7	170
4	D-TAMER V.3	x	x		n/a	n/a	x	x	169.45	145
5	D-TAMER V.4	x	x		n/a	n/a	x		98.15	175
6	D-TAMER V.5	x	x	x	n/a	n/a	x	x	111.05	135
7	HG-DAgger	n/a	x	x	n/a	x	n/a	n/a	107.6	175
8	HG-DAgger V.1	n/a	x		n/a	x	n/a	n/a	83	135
9	ICREATe V.1	x	x	x		x	x	x	193.85	75
10	ICREATe V.1	x	x	x		x	x		122.9	135
11	ICREATe V.1	x	x	x		x		x	198.15	40
12	ICREATe V.1	x	x	x		x			129.3	165
13	ICREATe V.2	x	x			x	x	x	173.6	125
14	ICREATe V.2	x	x			x	x		114.75	165
15	ICREATe	x	x	x	x	x	x	x	**199**.**5**	30
16	ICREATe	x	x	x	x	x	x		**193**	105
17	ICREATe	x	x	x	x	x		x	**196**.**5**	40
18	ICREATe	x	x	x	x	x			**188**.**65**	175
19	CEILing	n/a	n/a	n/a	n/a	x	x	n/a	17.85	25
20	CEILing 3x	n/a	n/a	n/a	n/a	x	x	n/a	19.7	15

The values in bold are shown in the best cases

**Table 2:** Results of the simulated teacher experiments with the Skiing environment

		Algorithmic variable				Algorithmic or Oracle variable		Oracle variable	Convergence	
#	Method	AQ	EQ	Episodic update	Passive reward	Corrections	Evaluations	No mistakes in AQ	Return	Episodes
1	D-TAMER	n/a	n/a	n/a	n/a	n/a	x	n/a	− 11364.25	60
2	HG-DAgger	n/a	x	x	n/a	x		n/a	− 9539.7	35
3	ICREATe V.1	x	x	x		x	x	x	− 10200	45
4	ICREATe V.1	x	x	x		x	x		− 10354.5	55
5	ICREATe V.1	x	x	x		x		x	− 8324.7	70
6	ICREATe V.1	x	x	x		x			− 8609.3	70
7	ICREATe	x	x	x	x	x	x	x	**− 6178.8**	45
8	ICREATe	x	x	x	x	x	x		**− 9307.75**	55
9	ICREATe	x	x	x	x	x		x	**− 5417.4**	40
10	ICREATe	x	x	x	x	x			**− 6622.6**	70
11	CEILing	n/a	n/a	n/a	n/a	x	x	n/a	− 28179.85	3
12	CEILing 3x	n/a	n/a	n/a	n/a	x	x	n/a	− 27172.5	40

The values in bold are shown in the best cases

**Table 3:** Results of the simulated teacher experiments with the Pong environment

		Algorithmic variable				Algorithmic or Oracle variable		Oracle variable	Convergence	
#	Method	AQ	EQ	Episodic update	Passive reward	Corrections	Evaluations	No mistakes in AQ	Return	Episodes
1	D-TAMER	n/a	n/a	n/a	n/a	n/a	x	n/a	− 16.6	120
2	HG-DAgger	n/a	x	x	n/a	x		n/a	4.8	130
3	ICREATe V.1	x	x	x		x	x	x	8.15	120
4	ICREATe V.1	x	x	x		x	x		5.5	135
5	ICREATe V.1	x	x	x		x		x	13.85	105
6	ICREATe V.1	x	x	x		x			12.45	115
7	ICREATe	x	x	x	x	x	x	x	**7**.**6**	135
8	ICREATe	x	x	x	x	x	x		**4**.**9**	110
9	ICREATe	x	x	x	x	x		x	**15**.**2**	65
10	ICREATe	x	x	x	x	x			**16**.**4**	90
11	CEILing	n/a	n/a	n/a	n/a	x	x	n/a	− 17.65	100
12	CEILing 3x	n/a	n/a	n/a	n/a	x	x	n/a	− 13.25	115

The values in bold are shown in the best cases

**Table 4:** Results of the simulated teacher experiments with the Enduro environment

		Algorithmic variable				Algorithmic or Oracle variable		Oracle variable	Convergence	
#	Method	AQ	EQ	Episodic update	Passive reward	Corrections	Evaluations	No mistakes in AQ	Return	Episodes
1	D-TAMER	n/a	n/a	n/a	n/a	n/a	x	n/a	25.4	75
2	HG-DAgger	n/a	x	x	n/a	x		n/a	79.3	45
3	ICREATe V.1	x	x	x		x	x	x	52.2	120
4	ICREATe V.1	x	x	x		x	x		46.8	85
5	ICREATe V.1	x	x	x		x		x	93.3	115
6	ICREATe V.1	x	x	x		x			84.9	55
7	ICREATe	x	x	x	x	x	x	x	**69**.**1**	100
8	ICREATe	x	x	x	x	x	x		**57**.**7**	80
9	ICREATe	x	x	x	x	x		x	**114**.**2**	105
10	ICREATe	x	x	x	x	x			**99**.**5**	120
11	CEILing	n/a	n/a	n/a	n/a	x	x	n/a	11.4	55
12	CEILing 3x	n/a	n/a	n/a	n/a	x	x	n/a	36.9	80

The values in bold are shown in the best cases

*Uncertainty evolution:* As mentioned in Sect. 4.1.1, the epistemic uncertainty is high at the beginning of the learning process because there are not enough data to describe all the possible situations, but it decreases over time, with more data collection, whereas the aleatoric uncertainty is null at the beginning and starts to increase when more and more noisy data are collected, as in this case—because we know the collected data have a high rate of mistakes.

Although it was mentioned that aleatoric uncertainty is not eliminated with more data (Sect. 2.3), with the proposed strategy of predicting mistakes (Sect. 3.2.2), the queries based on this uncertainty could actively obtain more data that unbalances the prediction toward the most demonstrated action out of the set of contradictory feedback related to a specific state. Therefore, the average prediction error of that state is reduced, and then it could be considered more certain.

**Fig. 6:**
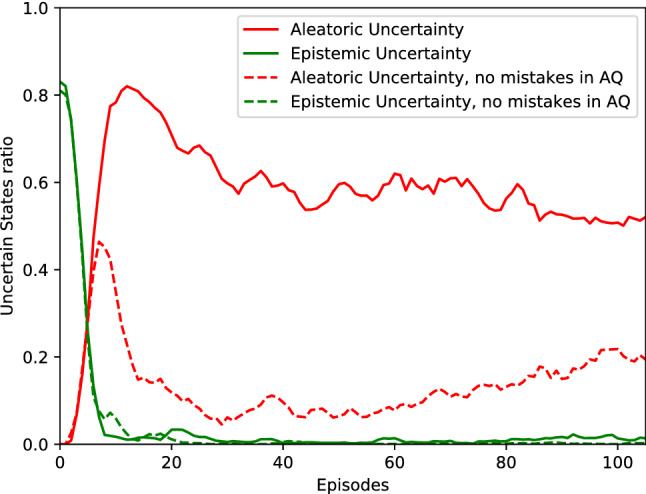
Evolution of the uncertain states ratio through the learning process

Figure 6 shows how the uncertain states ratio evolves through the episodes while learning in the CartPole environment. Both cases with mistakes and no mistakes during aleatoric uncertainty-based queries are depicted. The epistemic uncertainty is reduced in both cases as expected, while the aleatoric uncertainty increases after some feedback is collected, but is reduced later on with the data collected via active queries, especially if the response to the queries is less noisy (or not noisy in the case of “no mistakes in AQ”).

However, even though the teacher does not reduce the rate of mistakes during any query, the active queries can help to improve the balance of the contradictory data. For instance, as in the case of these simulated teacher experiments, if the teacher gives noisy feedback $40\%$ of the time, $40\%$ of the state space can have obtained contradictory feedback, while the remaining $60\%$ has correct demonstrations. Then, there will be aleatoric uncertainty-based queries in $40\%$ of the states, wherein the teacher again will provide $60\%$ of correct feedback, i.e., $24\%$ ($60\%$ of the $40\%$) would have contradictory samples that are biased toward the right demonstration, while only $16\%$ of the states ($40\%$ of the $40\%$) would remain receiving incorrect feedback, reducing the balance toward incorrect demonstrations that otherwise would be $40\%$ without active learning.

#### User study

After running the user study, the average of all the subjective scores along with the objective measures is introduced in Table 5. The SUS score ranges from 0 to 100, while the Usefulness and Satisfaction scores of the Acceptance scale range between − 2 and 2. In both cases, a higher score means a better experience for the user, at least in the domain the score intends to measure. In the case of the TLX questions, the scores also range from 0 to 100; however, high values are related to high workloads of the evaluated systems, and it is desirable to have them as low as possible.

The SUS score is in a similar range for all the evaluated cases, where the variations AQ, EAQ, and EAQPR got the highest score, i.e., all the cases using aleatoric uncertainty-based queries scored slightly more usable than only using epistemic uncertainty or no queries at all. In the results of the acceptance scale, the Usefulness scale shows a similar trend, having the systems using aleatoric uncertainty queries rating as the most useful (matching the two usefulness measures), however, in this case, the passive learners rated way lower, while the systems using only epistemic queries seem to be more useful than the passive learners, but not as much as the ones using the aleatoric uncertainty. The satisfaction scale shows again that active learners get better results, especially if they use the aleatoric uncertainty, although participants found that combining both uncertainties is slightly more irritating than only using queries in situations of ambiguity (as with AQ).

The results of the raw TLX questionnaire (without merging each question rate into one unified score) show a similar trend, having the passive learner with the highest workload, followed by the active learner EQ (only epistemic), and the ablations incorporating the aleatoric uncertainties being the least demanding. Only the physical demand was a point not providing any information because each of the participants rated all the ablations with a very similar rate, having variance only between participants. This result is expected since the physical activity is limited to moving a few fingers, and it is the same for all the cases.

In general, the use of positive rewarding did not show a major impact on the subjective measures of usefulness and workload; however, it did have an impact on the convergence of the learning process, with improvement of the policy performance and reduction of the number of interactions the participants had to do. Since the participants were not observing the score the agent obtains at the end of the episode (it is not printed by the environment on the screen as in other games), they could perceive only large policy improvements. Therefore, the lack of perception of improvement the passive rewarding obtains in the final performance, as it could be seen comparing EAQPR with EAQ, has its influence on the subjective scores.

However, in the satisfaction scale, it can be seen that the passive rewarding in EAQPR obtained an increment of $16\%$ with respect to not using it (EAQ). Although, these two ablations did not get the highest satisfaction, as it was mentioned before.

**Table 5:** Results of user study

Ablation			PL	EQ	AQ	EAQ	EAQPR
Subjective measures	SUS		83.21	84.82	**88**.**75**	**88**.**93**	**89**.**82**
	Acceptance scale	Usefulness	0.64	1.19	**1**.**47**	**1**.**50**	**1**.**51**
		Satisfaction	0.43	0.66	**1**.**05**	0.77	0.89
	NASA-TLX	Mental demand	68.21	58.93	**44**.**64**	**42**.**50**	**41**.**43**
		Physical demand	10.36	10.71	10.71	10.36	10.36
		Temporal demand	50.36	35.71	33.21	**24**.**29**	**26**.**79**
		I-Performance	42.50	32.50	**27**.**14**	**25**.**00**	**26**.**79**
		Effort	56.79	41.43	**36**.**07**	**33**.**93**	**32**.**14**
		Frustration level	42.86	35.00	29.29	**25**.**71**	**25**.**36**
Objective measures	Performance		− 15387.21	− 10872.00	− 8553.64	− 7752.35	**− 7034.42**
	Feedback instances		14472.71	13196.07	12049.29	10431.64	**9239**.**57**

The cases with the highest performances according to each index are shown in bold

#### Results in real robot environments

**Fig. 7:**
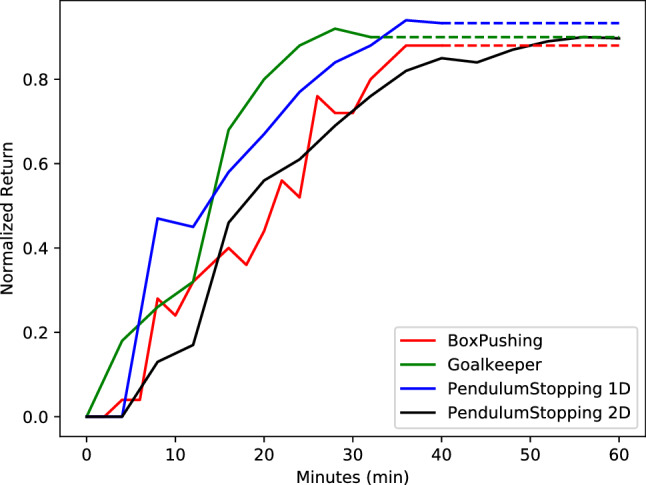
Evolution of the uncertain states ratio through the learning process

The tasks used for validating ICREATe with the real robot, feature low-dimensional state spaces, but not so simple dynamics, which are even fast for human teachers as in the case of the pendulum stopping tasks. In Fig. 7, the learning curve for these tasks is plotted with normalized objective functions, depicting that it is possible to train complex dynamic tasks, with robots in the physical world within a few minutes using ICREATe. The simplest task was the goalkeeper since the interaction of the robot and the object does not need to be precise, requiring around 20 min to achieve a good performance. The most demanding task was the PendulumStopping 2D, given the required fast reaction of the teacher, in order to compensate for the fast movements of the pendulum. The video^1^ of the paper shows the performance of these systems during and after learning.

## Conclusion

The proposed ICREATe is an IIL method that is epistemic and aleatoric uncertainty aware, features that are convenient for improving the data that composes the agents’ knowledge base, when the system is either lacking data, or having contradictory data. Additionally, ICREATe lets the user teach with evaluative or corrective feedback according to the user’s preference at any moment of the learning process. The algorithm smoothly combines both kinds of feedback to update the policy, and it can even model contradictions across the two kinds of feedback signals.

The experimental results showed how the active queries of ICREATe can help to considerably improve the learning performance under regimes of highly ambiguous teachers, while at the same time, the user study showed that they improve the overall teaching experience.

Given that most people take a teacher’s silence as positive feedback, the proposed method includes the positive rewarding assumption, which considers the teacher agreeing with a behavior when not “complaining.” This is only applied in situations of active queries because the teacher is more alert. This strategy allows to gather more relevant data without further effort.

Since it is known that corrective demonstrations are more informative than evaluations, the second objective of this paper was not to improve learning convergence through the combination of both kinds of feedback, but rather to improve the interaction flexibility of the teachers. However, the experiments showed that combining corrective feedback with implicit positive evaluative feedback has a positive impact in the data efficiency and the policy performance.

In general, when applying deep learning strategies for learning from massive amounts of data, aleatoric uncertainty can have a higher impact on the learning process [37]; however, IIL requires to collect the samples simultaneously during the learning process, sometimes starting from empty datasets, which makes epistemic uncertainty also relevant. Hence, making use of both uncertainties is a necessary consideration for learning with humans in the loop, especially when considering that we are not perfect oracles and we often do mistakes.

Nevertheless, further research should be carried out for having a method that allows users to combine smoothly any kind of human feedback. Moreover, ICREATe does not work for continuous action problems, but few feasible considerations are enough for adapting it to those kind of environments.

## Footnotes

1 https://youtu.be/_eS75eusTFQ.
